# Diversity of organ-specific plant transcriptomes

**DOI:** 10.3389/abp.2025.14609

**Published:** 2025-07-16

**Authors:** Michał Rurek

**Affiliations:** Department of Molecular and Cellular Biology, Institute of Molecular Biology and Biotechnology, Faculty of Biology, Adam Mickiewicz University, Poznań, Poland

**Keywords:** medicinal plant species, plant organs, plant transcriptome, RNA-seq, secondary metabolites

## Abstract

Plant transcriptomes comprise nuclear and organellar (mitochondrial and plastid) transcripts expressed by nuclear and organellar genomes, respectively. They are spatiotemporally shaped during development. The aim of this review was to summarize the most relevant transcriptomic responses in various plant organs and tissues in the developmental context. The dynamicity of organ- or tissue-specific transcriptomic responses was discussed based on the complexity and diversity of the recently characterized plant genomes and transcriptomes. Data were taken from high-throughput studies on numerous species, including model, crop and medicinal plant species. Vascular element transcriptomes as well as the root, leaf, flower and seed transcriptomes were exhaustively characterized. Transcriptomic alterations within various tissue and organ-specific transcriptomes employed various gene classes depending on the species, a given organ/tissue and the developmental stage. The specificity of organ-specific transcriptomes related to the over-representation of certain gene families and a plethora of transcription factors was focused. In addition, transcriptomes of medicinal plant species were characterized. The perspectives of medicinal plant species to synthesize valuable secondary metabolites (including quinones, carotenoids, phytoestrogens, terpenoids, steroids, flavonoids, phenolic derivatives, polysaccharides, glycosides, anthocyanins and macrocyclic peptides) were described based on organ transcriptomic patterns. Future research should be broadened by investigation of transcriptomes from field grown plants. Also, the potential of biomedical plants should be better revealed by genetic engineering and genome editing in further biotechnological applications.

## Introduction

Higher plants are characterized by the development of highly specialized tissues and the dominance of sporophyte over gametophyte (Kenrick and Crane, 1997; Forster et al., 2007). During life evolution, higher plants adapted to the environment in multiple aspects, including both developmental adaptations and adequate transcriptomic responses (Morris et al., 2018).

Complex plant transcriptomes, composed of RNA molecules of various origins, are shaped spatiotemporally during plant development. According to Imadi et al. (2015), plant transcriptome depends on an actively transcribed genome fraction, and which is required to respond to environmental cues in various tissues and organs. However, the transcriptomic dynamicity relies not only on the given tissue but also on sampling procedures. Tissue and organ-specific transcriptomes offer valuable information on the molecular processes that affect their functions, such as photosynthesis in leaves, or nutrient uptake in roots. Investigating tissue-specific transcriptomes, multiple specific genes and regulatory mechanisms that display unique attributes can be retrieved, allowing, for instance, agricultural improvements (Booth et al., 2022).

The development of high-throughput RNA-seq platforms with the elaborated meta-analyses and the subsequent decrease in sequencing costs, simplified plant transcriptomic studies. Currently they allow for the analysis of specific features of cells, tissues, or organs in various aspects (Rurek and Smolibowski, 2024). Notable factors that precisely regulate the transcriptional activity of a given biological system can also be characterized (Zhang, 2019; Tyagi et al., 2022). Recently, the relevance of transcriptomic responses from single cell or single nuclei RNA-seq (scRNA-seq and snRNA-seq, respectively) has been greatly expanded by organ-specific studies. They allow us to characterize numerous gene pathways and networks expressed in a cell-specific manner (Kortz et al., 2019; Kao et al., 2021; Picard et al., 2021; Lee et al., 2023). It should be noted that in the meantime various assays dedicated to single-cell sampling have been developed, including fluorescence-activated cell sorting (FACS) and laser-capture microdissection (LCM). LCM allows isolation of cells from tissues containing various cell types (Nelson et al., 2006; Galbraith, 2012; Gautam and Sarkar, 2015).

We have already presented dynamic transcriptomic replies to various stressors, including participation of specific gene families and transcription factors (TFs) (Rurek and Smolibowski, 2024). In the current review, the diversity of transcriptomic responses in various plant organs and tissues in development will be widely summarized. Focus will be made on data from high-throughput approaches, including various RNA-seq platforms, single cell/nuclei transcriptomics and methods employing expression slides. The transcriptomic patterns from model, crop as well as medicinal plant species will be discussed. It should be underlined that genomes of non-model plant species may be particularly complex and the transcriptomic data can bypass genomic gaps (Hirsch and Robin Buell, 2013). The dynamic transcriptomic patterns (including organellar responses) will be summarized based on studies on the biogenesis of vascular tissues, seeds, vegetative (including leaves and roots) as well as flowers. The potential of medicinal plant species for secondary metabolite synthesis will be also discussed based on valuable transcriptomic analyses. In general, data discussed in the current review would complement Rurek and Smolibowski (2024) study by summarizing organ- and tissue-specific transcriptomic responses across plant development.

## The variety of higher plant genomes and transcriptomes contributes to the complexity of transcriptomic responsiveness

Before investigating a transcriptomic diversity across various plant organs and tissues, its dependance on the genomic level should be characterized first.

The plant genome contains all DNA molecules in the cell. It is composed of the nuclear and organellar genomes (plastome and mitogenome referred to chloroplast and mitochondrial DNA, respectively). Land plants contain the most conserved nuclear genomes by size, unlike plastids and mitochondria. For example, a carnivorous plant *Genlisea aurea* possess one of the smallest nuclear genomes (43.3 Mbps), while *Paris japonica* contains one of the largest ones (up to 150 Gbps) (Supplementary Table S1). Currently, the recurrent whole genome duplications (WGDs), depending on the polyploidy and the transposition frequency are thought to be responsible for the large-scale variation of nuclear genome size in land plants. Although duplication and alterations in chromosome numbers obviously explain the increasing genome size, transposon expansion results even in the greatest variation in the genome size. The relationship between genome size and transposon content is generally linear (Kress et al., 2022). The size of the nuclear genome of plants correlates also with the nutrient deficiency, when species with larger genomes are unable to dominate in ecosystems (Pellicer et al., 2018).

Plastids and mitochondria originated from the endosymbiotic engulfing and maintenance of single-celled organisms in the early eukaryotic cell. In some taxa, serial endosymbiosis explains the complex origins of their plastids (Margulis, 1970; McFadden, 2001). The mitochondrial ancestors were probably γ-proteobacteria, while the plastid ones- cyanobacteria-similar organisms. Most organellar genes underwent an evolutionary transfer to the nucleus. Nowadays, organellar DNA insertions (nuclear-mitochondrial or nuclear-plastid insertions) can be also found in nuclear genomes (Rockwell et al., 2014). Genome-containing organelles thus exhibit a particularly complex pattern of their biogenesis, depending on concerted regulated expression of nuclear and organellar genes (Best et al., 2020a; Dobrogojski et al., 2020).

Land plant plastome is generally conserved in length and organization (120–180 kbps in size) (Provan et al., 2001; Asaf et al., 2017). The size of the Arabidopsis plastome (compared with nuclear genome of 119.1 Mbps) is 154,478 bps, when *P. japonica*, the owner of the largest land plant nuclear genome (150 Gbps), has a plastome of 155,957 bps only. This gives a difference of Arabidopsis and *P. japonica* plastomes of about 1,500 bps (Supplementary Table S1). Land plant plastomes encode a limited number of proteins involved in photosynthesis, transcription, translation and plastid signaling as well as diverse rRNA, tRNA and ncRNA molecules (Daniell et al., 2016; Rurek, 2016). Multiple nuclear factors regulate the expression of plastid genes (Barkan and Goldschmidt-Clermont, 2000).

Compared to plastome, land plant mitogenomes range from 208 kbps for white mustard (*Brassica hirta*), 366 kbps for Arabidopsis, up to 11.3 Mbps in size in *Silene* species (Supplementary Table S1), while the gene number (approx. 60 genes) remain relatively stable (Logan, 2006; Sloan D. B. et al., 2012a; Sloan D. et al., 2012b). However, the size of mitogenome copies can vary in the same species, for example, the potato (*Solanum tuberosum*) mitogenome contains few molecules ranging from 49,171 bps to 297,014 bps in size (Cho et al., 2017) (Supplementary Table S1). Higher plant mitogenomes are therefore particularly large. They display a complex structure (a mixture of circular, linear and concatemeric forms), which probably optimized seed germination in novel ecosystems in the Palaeophytic era. Interestingly, plant mitogenome structure and expression affect mitochondrial biogenesis under organogenesis (Best et al., 2020a).

Plant transcriptome is set of various RNA molecules spatiotemporally regulated. The transcriptome capacity extends the genome size. For instance, the capacity of maize (*Zea mays*) total transcriptome was estimated to 97 Mbps, which consists only 4% of the nuclear genome size (Schneider and Dekker, 2012). To date, Best et al. (2020b) have published maps of Arabidopsis and cauliflower (*Brassica oleracea* var. *botrytis*) mitochondrial transcriptomes. Arabidopsis and cauliflower mitogenomes encode 28 and 33 protein-coding genes, 3 and 3 rRNAs, 22 and 18 tRNAs, and cover approximately 85 and 35 ORFs of >100 amino acid residues, respectively. In addition to rRNAs, numerous tRNAs and ncRNA molecules (Rurek, 2016), plant mitogenomes encode OXPHOS proteins, ATP synthase subunits, mitoribosomal proteins, few proteins for cyt. *c* biogenesis and the twin-arginine translocation protein (Tat). At least 42 and 33 transcription units were found by RNA-seq of Arabidopsis and cauliflower mitogenomes, respectively. The expression of several Arabidopsis mitogenes leads to the formation of mono or bicistronic transcripts. Various open reading frames within the same polycistronic transcript are diversely expressed within post-transcriptional RNA processing (Cahoon et al., 2017; Best et al., 2020a).

To sum up, the diversity of plant genomes and transcriptomes belongs to important factors that contribute to the plasticity of transcriptomic dynamic response under development.

## Transcriptomes of selected plant organs and tissues

**FIGURE 1:**
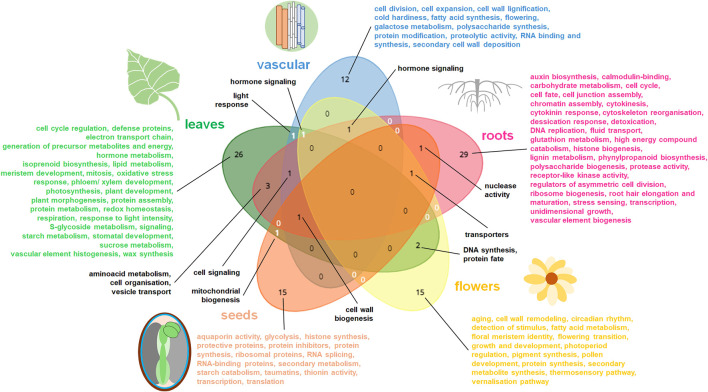
The summary of gene functions (GO: terms) for the most relevant, upregulated gene families in diverse plant organs. The most common and specific organo-specific transcriptomic replies from cited literature are shown. The data specific for distinct organs were presented in diverse colors on Venn diagram. Venn diagrams for five datasets (representing roots, vascular elements, leaves, flowers and seeds) were drawn using tool from https://www.biotools.fr/misc/venny.

**FIGURE 2:**
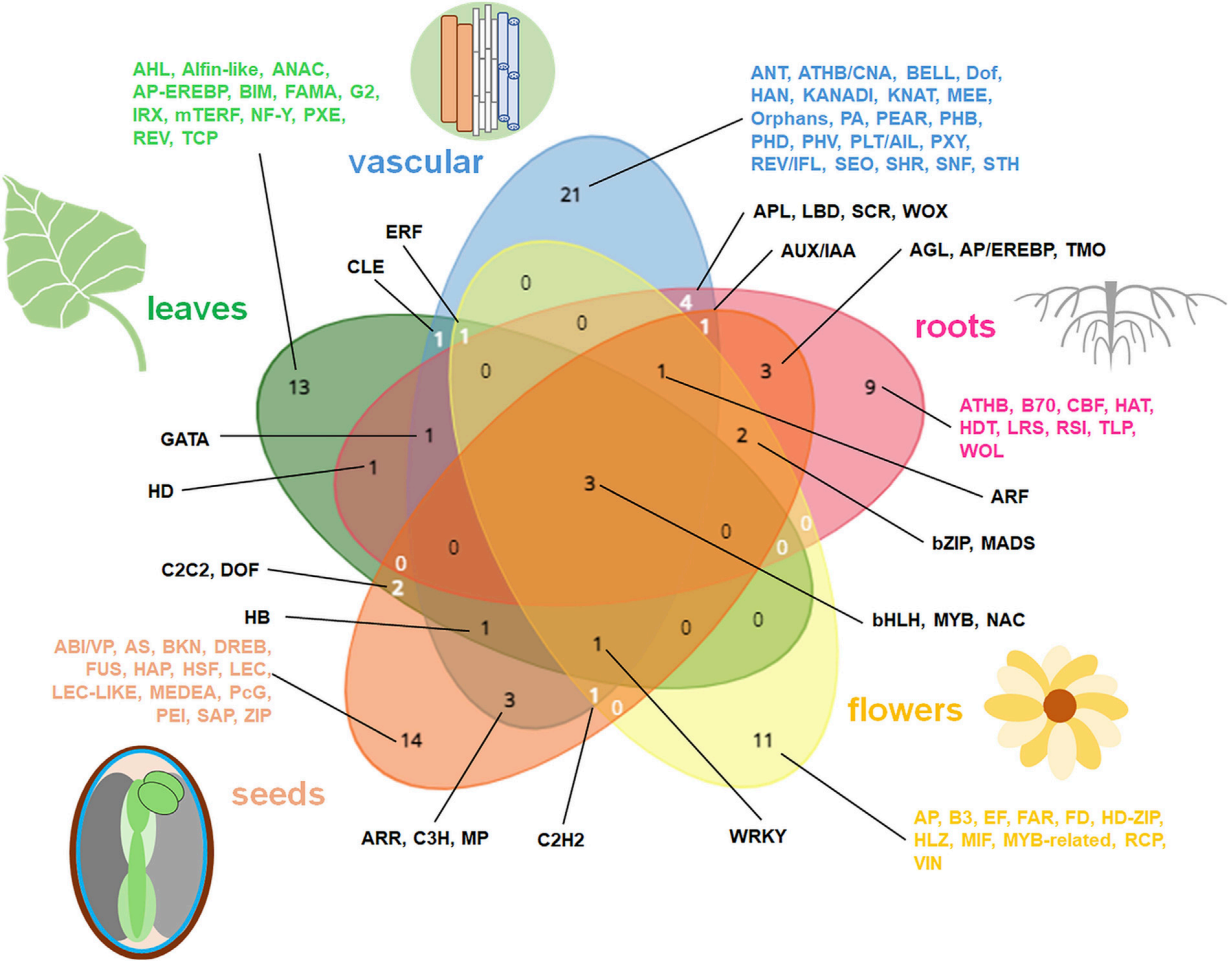
The summary of the most important TFs in diverse plant organs. The most common and specific organo-specific TFs from cited literature are shown. The data specific for distinct organs were presented in diverse colors on Venn diagram. Venn diagrams for five datasets (representing roots, vascular elements, leaves, flowers and seeds) were drawn using tool from https://www.biotools.fr/misc/venny.

**FIGURE 3:**
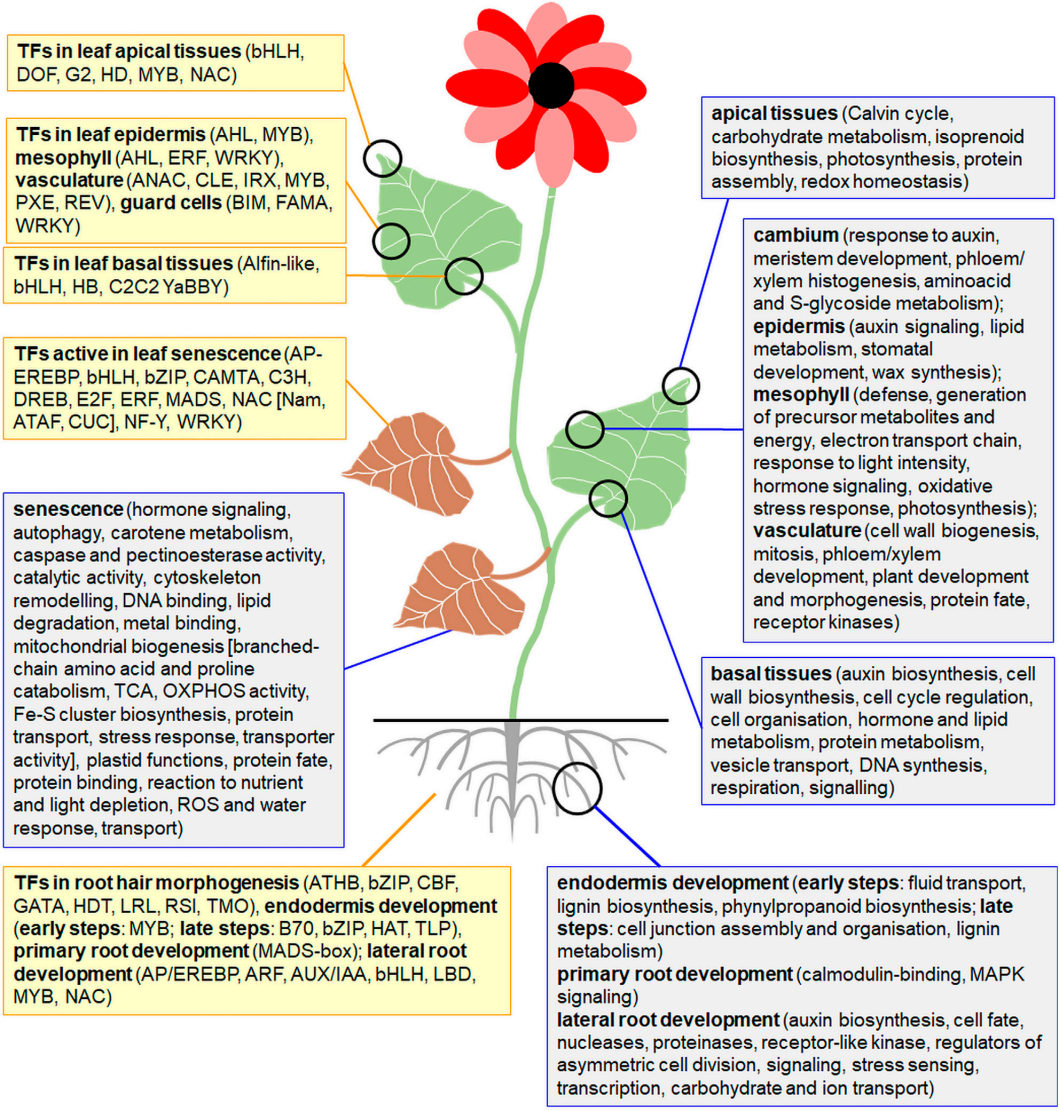
Tissue- and developmentally specific expression patterns in roots and leaves. The functional GO: terms for the most relevant, upregulated gene families and the most important TFs in various tissues of roots and leaves and across the diverse developmental stages were shown in separate boxes.

**FIGURE 4:**
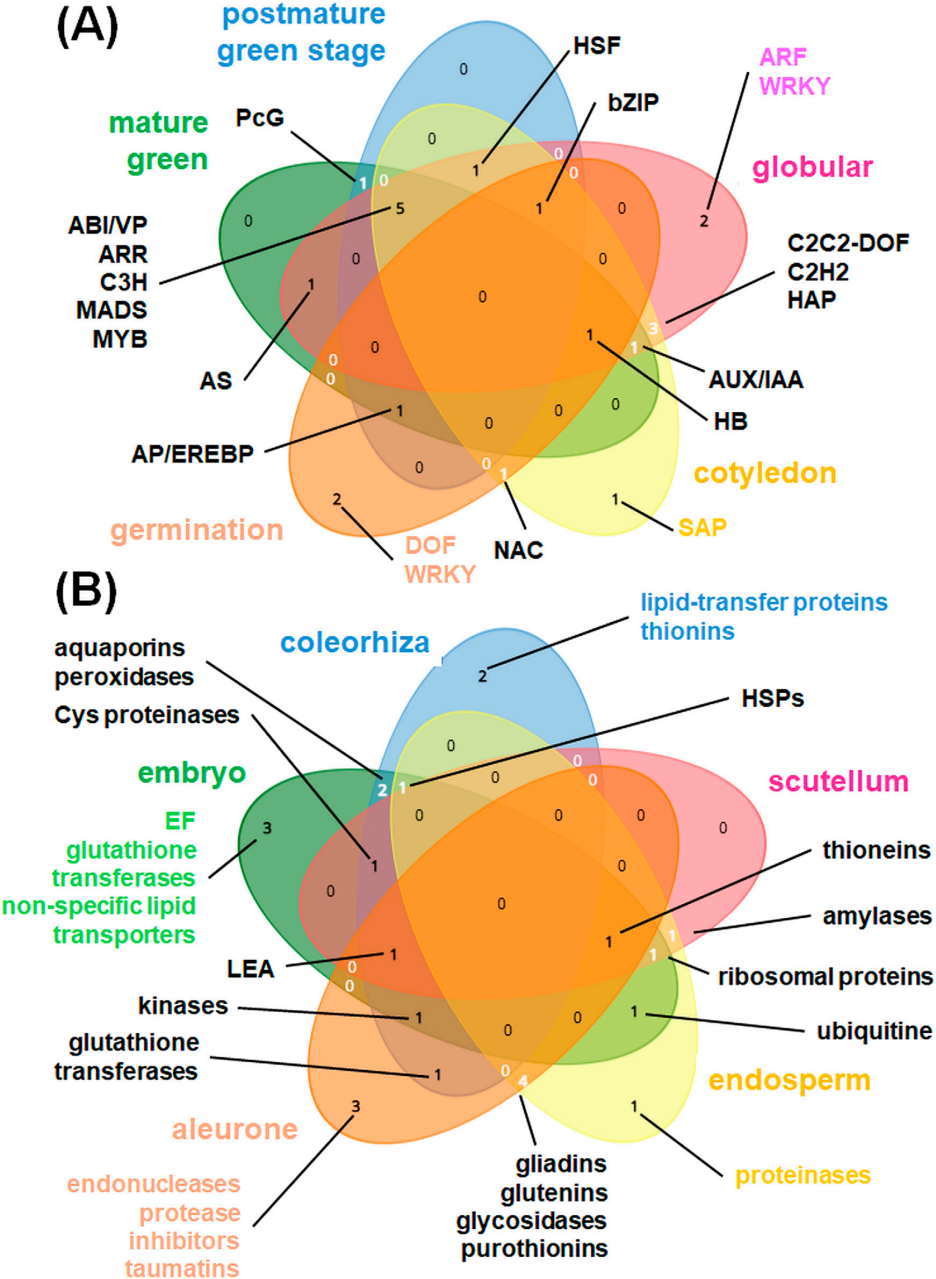
Tissue- and developmentally specific expression patterns in seeds. Venn diagrams showing **(A)** most abundant TFs active during various seed developmental stages (cotyledon, germination, globular, mature green, postmature green); **(B)** mostly enriched protein families coded by DEGs in coleorhiza, embryo, scutellum, endosperm as well as aleurone layer. Venn diagrams for five datasets at each time were drawn using tool from https://www.biotools.fr/misc/venny.

The specialization of individual plant organs to perform specific functions is a consequence of differences in the gene expression patterns between those organs (Huang et al., 2016). Details on affected genes from developmental and organo-specific studies are given in Supplementary Table S2. Figures 1, 2 summarize the most relevant gene functional classes and the most notable TFs in diverse plant organs. Further details on tissue- and developmentally specific expression patterns within selected plant organs (roots, leaves, seeds) were shown in Figures 3, 4.

### Vascular element transcriptomes

Vascular element transcriptomes belong to extensively studied tissue-specific transcriptomes. During the primary growth of stems and roots, procambium derived from apical meristems differentiates into xylem and phloem. Precursor xylem cells form xylem crumb cells or fibers. In the vascular bundle, xylem, procambium and phloem cells show dorsoventral polarization. Xylem is located on the dorsal (adaxial) side, phloem occurs on the ventral (axial) side, and procambium- between the phloem and xylem (Wang and Dixon, 2012; Růžička et al., 2015).

Cambium transcriptome is actively remodeled under dormancy, reactivation and activity stages. Multiple genes govern cambial cell division and expansion, and cell wall component biogenesis. Various MYB and NAC TFs regulate gene expression especially during lignin formation (Du et al., 2024). *WOX* and *PXY* genes for respective TFs belong also to important regulators of cambium biogenesis (Chen et al., 2021; Zhou et al., 2024; Sheng et al., 2025). Studying poplar *P. tremula* transcriptome Chen et al. (2021) characterized cambium marker genes. They were: *MP* gene for auxin-responsive TF, *PIN1* for auxin efflux transporter, cytokinin-responsive transcription factor gene (*ANT*) and positive regulatory peptide of cambial activity (*CLE47*). Moreover, HD-ZIP III TFs (including *Ptr*HB4, *Ptr*HB7,and *Ptr*HB8) and *Pag*GRF15, a growth-regulating factor are also important for poplar cambium vascularization (Chen et al., 2021; Zhou et al., 2024). Overexpression of *Pag*GRF15 results in the decreased plant height and internode number and by reduction of phloem and expansion of xylem. Those effects indicate that GRF15 belongs to positive regulators of xylem differentiation, which represses *WOX* and *PXY* gene activity and stimulates *GID1.3* expression (Zhou et al., 2024). Multiple TF genes associated with phloem and cambium development in *Citrus macrophylla* trees were also active under *Citrus tristeza virus* infection (Khalilzadeh et al., 2022).

In stems of poplar hybrid lines (*Populus alba x P. glandulosa*), rectangle-shaped procambium-like cells develop from procambium meristem and produce phloem cells, whereas fusiform-shaped cells (inside the cambium zone) are produced by fusiform metacambium meristem and differentiate into xylem. Meristem identity markers enriched in those cells included mainly genes for TFs, e.g., *ANT*, *CLE*, *WOX*, *SCR* and *SHR*. Phloem and xylem identity genes covered also genes for important TFs and receptor kinases, like *CLE*, *SMXL*, *BAM*, *Dof*, *APL* and *SEO* for phloem, and *WOX*, *PXY*, *ATHB* and *VND* genes in case of xylem. Genes for the secondary cell wall biosynthesis were also highly enriched (Du et al., 2023). In poplar stems, Li et al. (2023) used *WOX4* and *PXY* genes as cambium markers and *XCP1* and *XCP2* genes as vessel element markers. *ACL5* was xylem precursor marker and *SEOR1* and *CLE41* genes were phloem markers. In addition, genes for xylem (*ACL5, MP, LBD4, NPY2, BSK6, ARR4, LAC6*) and phloem precursor branch development (*CLE41, BAM3, KNAT1, PA, MEE32, GH9B13, PER73*) encode important developmental regulatory proteins. Furthermore, transcriptomes of *Eucalyptus urograndis* vascular cambium at four developmental stages were investigated by Liu et al. (2023). Differentially expressed genes (DEGs) for various proteins, including expansins, kinesins, cyclins, lignification enzymes and various TFs were highly upregulated.

Seasonal variations in transcriptomes of meristem cambium and derivative cells of Japanese cedar (*Cryptomeria japonica*) under development were also studied (Mishima et al., 2014). Multiple DEGs involved in xylem formation, e.g., genes associated with cell wall biogenesis (including *PAL*, *4CL*, *C4H*, *HCT*, *CCOAOMT* and *CCR* for lignification), carbohydrate metabolism and cold hardiness, were upregulated in spring (Supplementary Table S2). The activation of cell division and the induction of cell cycle genes are thus correlated in early stages of xylogenesis. Various TFs active in *C. japonica* cambium cells covered C3H, NAC, PHD, AP2-EREB, HB, bHLH, SNF2, WRKY, C2H2, MYB and Orphans proteins (Mishima et al., 2014). *WOX* and *PXY* gene co-expression associated with the June peak of vascular system development in *Camellia chekiangoleosa* stems (Sheng et al., 2025).

Phloem elements are frequently enriched in highly upregulated DEGs for RNA polymerases subunits, enzymes of galactose metabolism, polysaccharide and fibrous element synthesis. In contrast, upregulated genes involved in fatty acid synthesis are expressed mostly in xylem (Weber, 2002; Chen et al., 2014). In phloem-associated cells of potato (*Solanum tuberosum*) petioles, genes for PPR proteins, kinase receptors, UDP-glucuronic acid decarboxylase were uniquely expressed (Supplementary Table S2) (Lin et al., 2015). Multiple TFs, including AUX/IAA, ERF, bZIP, BELL, HD, NAC and WRKY regulated gene expression in phloem-associated cells of potato leaf petioles and plant stems. Mutations in *REV/IFL1*, *PHB* and *PHV* genes have been shown to affect the organization of vascular tissues that contain phloem, which surrounds xylem. However, when *KANADI* genes (e.g., *KAN1*, *KAN2*, *KAN3*) are mutated, xylem surrounds phloem (Eshed et al., 2001; Dinneny and Yanofsky, 2004; Eshed et al., 2004). HD-ZIP III (e.g., ATHB8, ATHB15/CNA, PHV, PHB, REV/IFL1 TFs) and KANADI TFs act antagonistically on the xylem and phloem patterning, respectively. Other genes, coding for APL and MYB family TFs, are also necessary for the phloem specification (Bonke et al., 2003). Using scRNA-seq, Roszak et al. (2021) studied participation of PEAR proteins which mediate early asymmetric divisions during phloem cell biogenesis and in laterally adjacent procambium cells. They promote further activity of *APL* gene for the phloem regulator protein as well as protophloem, metaphloem and procambium differentiation. Gradient of PLT/AIL and HAN TFs affects differentiation of protophloem sieve elements by repressing *APL* transcription.

## Root and leaf transcriptomes

### Generally responsive DEGs and marker genes

Roots store high-energy compounds, e.g., complex carbohydrates, while leaves are photosynthetically active (Mason et al., 2020; Schmidt-Rohr, 2021). Those functions count for main differences in root and leaf transcriptomes (Figures 1, 2). For instance, in sugarcane (*Saccharum* spp. *hybrids*) and *Atractylodes lancea* transcriptomes, more DEGs for photosynthetic proteins (including Rubisco and chlorophyll *a-b* binding proteins, indicating for plastid relevance) and for signal transduction were expressed in leaves. Leaf transcriptomes are enriched in genes coding photosynthetic proteins, cell redox homeostasis and hormone signaling pathways (Figure 1) (Xanthopoulou et al., 2021). Among proteins upregulated in leaves, most of mTERF TFs are organelle targeted proteins. In contrast, root transcriptomes of both species were enriched with transcripts for polysaccharide biogenesis, amino acid metabolism, catabolism of high energy compounds and hormonal signaling (Supplementary Table S2) (Huang et al., 2016; Mason et al., 2022). Interestingly, leaf and root transcriptomes from the same cell layers possess similar features, with some exceptions. A set of 40 marker genes for 13 leaf cell types and at least 15 markers for 5 root cell types included also some genes for plastid (Rubisco small subunits, chlorophyll a/b binding protein) and mitochondrial (formate dehydrogenase) proteins. *RBCS4* and *Lhcb1.1* genes were expressed in mesophyll cells of diverse origin. *WOX4* and *CDKB2* genes were active in vascular initial cells and leaf primordia cells (Wang et al., 2021).

In recent years, detailed analysis of root and leaf transcriptomes often employed scRNA-seq (Efroni et al., 2016; Denyer et al., 2019; Shulse et al., 2019; Liu H. et al., 2021; Wang et al., 2021; Tenorio Berrío et al., 2022; Guo et al., 2025).

### Root transcriptomes

High-resolution expression atlas of Arabidopsis roots allowed for the fine resolution of marker genes by defining unique clusters for all major cell types. Expression of some nuclear genes was specific to those clusters. Markers specific to columella covered *ATL6* for RING-type E3 ubiquitin related enzyme, *PLT2/PLT3* for patterning root cells*, COBL2* for glycosylphosphatidylinositol-anchored protein and *NCED2* for 9-cis-epoxycarotenoid dioxygenase (all in columella). *SHR* gene for SCARECROW-like TF was expressed in endodermis, and *APL* gene for FE protein - in phloem and pericycle. In addition, *COBL9* gene was active in trichoblasts and *GL2* (coding HD protein) in atrichoblasts. Expression profiles of 239 TFs were distinctive, including various TFs regulating root hair biogenesis (Denyer et al., 2019). In general, almost 800 genes encoding proteins for cell junction biogenesis, polysaccharide synthesis, stress response, transport and protective functions were active for the endodermis biogenesis in Arabidopsis; diverse TFs regulated early and late stages of this process (Figure 3) (Shulse et al., 2019).

Known roots tissue markers are *GL2* and *WER* for MYB-like TF in non-hair cells, *SCR* in endodermis, *AGL42* and *WOX5* (for HB protein) in quiescent center cells, *APL*, *MYB46*, *SUC2* (encoding sucrose transporter) and *WOL* for two-component signal transducer in stele cells and *COBL9* for hair cells. Shulse et al. (2019) proposed a set of 17 additional nuclear genetic markers for diverse cell subpopulations. Markers for the lateral root organogenesis included *PASPA3*, *BFN1*, *SMB* and *RCP1* genes for proteinase family protein, bifunctional nuclease, NAC family TF and the maltose transporter, respectively (Figure 3) (Kortz et al., 2019).

Genes expressed in primary and lateral roots of Summer squash (*Cucurbita pepo*) highly overlapped. However, *ACS* gene for stress-sensing and signaling and genes for Cu and nitrate transporters belong to the lateral root-specific genes and genes for calmodulin-binding proteins and for MAPK signaling- for primary root specific genes (Xanthopoulou et al., 2021). The primary root development is also controlled by multiple factors, including MADS-box proteins that repress root growth and control meristem features, cell division rate and the length of elongated cells (Figure 3) (Castañón-Suárez et al., 2024).

The pattern of root regeneration after root tip excision followed embryonic patterning and was not driven by the initiation program of lateral root biogenesis. In fact, the transcriptome of regenerating cells prior to stem cell activation resembled that of embryonic root progenitor cells. The activity of endodermal *SCR* promoter appeared important for regeneration of new endodermis and lateral root caps (Efroni et al., 2016).

Some genes for plant root development were edited with a very promising effects. For instance, *ARG* gene for arginase which targets NO synthase was knocked out by Wang et al. (2017). Those attempts resulted in the increase of lateral root number and total root surface which improved root development by more efficient water and nutrient uptake from the soil. RNA virus-mediated delivery of sgRNA along with the cytokinin biosynthesis gene, isopentenyl transferase to potato (*Solanum tuberosum*) axillary meristems was carried out by Liu et al. (2024). In result, abundant gene-edited shoots displayed normal phenotype. This approach overcame challenges in virus-induced gene editing strategy to dicot crop species.

### Transcriptomes of leaf tissues and senescent leaves

In Arabidopsis leaf transcriptome, a number of tissue-specific genes decreased from the leaf vasculature to the epidermis and mesophyll, indicating their various sensitivity to transcriptional responsiveness. Multiple nuclear genes specific to biogenesis of epidermis (*KCS, GPAT, CER, LACS*, *MYB16, MYB30* genes for various enzymes and genes for MYB TFs), mesophyll (genes for ERF and WRKY TFs) and vasculature (*IRX*, *CLE*, *PXE* and *REV* genes as well as genes for ANAC and MYB TFs) were characterized (Berkowitz et al., 2021). Various classes of DEGs were upregulated in distinct zones of maize (*Zea mays*) leaves, for instance respiratory, cell wall biogenesis, and auxin and brassinosteroid signaling genes in basal tissues, while photosynthetic and sucrose transporter genes were expressed mainly in leaf tips (Figure 3).

Furthermore, genetic markers for transcriptomes of distinct cells from Arabidopsis leaves were proposed by Tenorio Berrío et al. (2022). The expression pattern of *PHYTOCYSTATIN1* (*CYS1*) and genes for plastid proteins delineated all vasculature- and mesophyll-derived (mostly photosynthetic) cells, respectively. In addition, nuclear genes: *EXTENSIN-LIKE PROTEIN* (*ELP*) and *GERMIN3* (*GER3*) were enriched in mesophyll, *PROLINE-RICH PROTEIN4* for a cell-wall protein–in epidermis and *WINDHOSE1* - in vasculature. Interestingly, flavonols and anthocyanins were synthesized mostly at the adaxial leaf side. Genes for glucosinolate metabolism enzymes differentiated diverse vasculature-derived cells. Transcriptomes of bundle sheath (genes for photosynthetic, respiratory and transport proteins) and mesophyll cells (genes for PSII, translation, secondary metabolism, and vesicle transport proteins) were also compared (Li et al., 2010; Tenorio Berrío et al., 2022).

As concerns mesophyll biogenesis, palisade cells are able to differentiate into spongy cells, while epidermal cells originate earlier than the primordial ones. Ectopically expressed AHL23, a nuclear TF belonging to proteins important for mesophyll and epidermis development, alleviated peanut (*Arachis hypogaea*) leaf growth. Five highly expressed marker genes for each of eight cell clusters coded, among others, ribosomal subunits (RPS, RPL), plastid (NDHJ, psbB) and mitochondrial (COX3) proteins (Liu H. et al., 2021). Such results highlight the relevance of expression patterns of genes for organellar proteins in leaf development. For instance, mutations in *PPR446* gene for chloroplast protein with 11 PPR domains or silencing of PPR466 expression affected leaf development (the appearance of *albino* phenotype; Zhao et al., 2021). Some conserved TFs were particularly highly accumulated in guard cells (Figure 3) (Lee et al., 2023).

The transcriptome of senescent Arabidopsis leaves became distinct from that of mature leaves (Breeze et al., 2011). During leaf senescence, genes for jasmonic acid (JA) and ethylene signaling, stress response, caspase and pectinoesterase activity, lipid degradation, cytoskeleton, metal binding and transport were upregulated while DEGs related to chlorophyll biogenesis, photosynthesis, cytokinin signaling, ribosome biogenesis, amino acid metabolism and cell cycle were downregulated. Some genes coding for plastid proteins (PSBQ and PSBP subunits of PSII, and CAROTENOID cleavage dioxygenase4) are also involved in carotenoid degradation in senescent leaves (Gonzalez-Jorge et al., 2013). Leaf senescence is also regulated by multiple TFs (Figure 3) (Breeze et al., 2011). Chrobok et al. (2016) performed the meta-analysis of Breeze et al. (2011) data and identified more than 1,000 genes for mitochondrial proteins active in leaves especially at the early senescence stages. The mitochondrial cluster was particularly enriched in genes for OXPHOS proteins, subunits of main mitochondrial importin, some transporters, auxin signaling, reaction to nutrient and light depletion, plastid functions, stress response, protein fate. As senescence progressed, genes for plastid proteins displayed similar patterns to the mitochondrial ones. The late stages of leaf senescence were enriched in genes active in branched amino acid and proline metabolism, which represented ancestral mitochondrial functions (Figure 3). Interestingly, chloroplasts size, but not quantity considerably declined, and mitochondria quantity decrease under leaf senescence. However, mitochondrial integrity as well as ATP production was substantially preserved during this process. Overall, Chrobok et al. (2016) highlights the relevance of mitochondrial metabolism to support the aminoacid and fatty acid catabolism in the senescence of Arabidopsis leaves.

Guo et al. (2025) generated Arabidopsis transcriptomic atlas from spatiotemporal snRNA-seq and proposed molecular markers to quantify cells. Thousands of senescence-associated genes (*SAG*s; especially at the late stages) were analyzed. Investigated markers covered early (*SAG13*) and late (*SAG12*) senescence genes as well as genes associated with Rubisco activity (*RBCS1A*) and chlorophyll catabolism (*NYE2*).

### Transcriptomes of generative organs

Flower morphology is species-specific. Flowering depends on pollination strategy, photoperiod, vernalization, phytohormone activity, thermosensing and aging-associated processes (Melzer et al., 2008; Kim et al., 2009; Mutasa-Gottgens and Hedden, 2009; Srikanth and Schmid, 2011; Liu et al., 2016). Despite the differences between flower organogenesis, the transition from the vegetative to the generative phase is tightly controlled (Huang et al., 2013).

In *Annona squamosa* transcriptome, upregulated genes for vernalization and photoperiod induction covered phytochrome (*PHY*) and cryptochrome (*CRY*), as well as early flowering (*EF1*, *EF3*) genes and *FIE* and *VIN3* genes (Liu et al., 2016). Regarding TFs important for flowering transition, the activity of MADS-box genes (e.g., *AGL*, *FUL*, *SOC*) and MADS proteins (including SOC1 and FUL) is notable (Figures 1, 2). MADS-box proteins, including VRN1, FUL2 and FUL3 play a role in the differentiation of the upper spikelet ridge (formed by the inflorescence meristem) and HD-ZIP III TF (WPS1-like protein) controls spikelet pairing in wheat (*Triticum aestivum*) (Li et al., 2019; Zhang et al., 2022). Other genes affecting flower morphology and size (by promoting petal expansion) belong to AUX/IAA family with expression repressed by ethylene (Jia et al., 2022; Wang et al., 2024b).

In early flowering stages, hormone signaling-related and stress-responsive genes are expressed. Genes related to floral organ development are upregulated generally in flower determination and maturation stages (Figure 1) (Jiao et al., 2019). Disturbances in the expression pattern of those genes contributed to delayed flower formation, despite the unaffected photoperiod (Melzer et al., 2008). In common walnut (*Juglans regia*) under flower bud development various genes for DNA replication and flavonoid synthesis were stage-specifically expressed. The circadian rhythm plant pathway dominated at the initial stages of apical meristem transformation into pistil primordium and covered genes homologous to Arabidopsis circadian clock genes *LHY*, *PRR, FKF* and *GI* (Ma et al., 2021). At the petal formation stage, genes coding for enzymes for carotenoids or anthocyanins, were significantly upregulated in diverse *Achimenes* species (Roberts and Roalson, 2017). Some flower buds go through dormancy phase; Prudencio et al. (2021) studied early and late flowering genes in endodormant and ecodormant almond (*Prunus dulcis*) flower buds related with proteins for carbohydrate metabolism and cell wall remodeling (endoglucosidases, glucanases, galactosyltransferases), transmembrane transport (aquaporins, sugar transporters), lipoxygenases, hormonal signaling (ABA biosynthesis), pollen development as well as MADS-box and HLZ proteins (Supplementary Table S2).

Flower petal colorization is an important step, where expression of multiple genes for various pigment (e.g., flavonoid) synthesis is turned on. It depends on the activity of regulatory proteins, including mini zinc-finger protein (MIF1), as was shown for *Gentiana triflora* flowers. In faint-blue plants, *MIF1* gene is differentially expressed and determines gentian color intensity (Tasaki et al., 2022). Strikingly, enhanced expression of some flavonoid biogenesis genes resulted in decreased anthocyanin pigmentation in *Chrysanthemum* flowers under thermal stress (Shi et al., 2022). In *Lysimachia arvensis* with blue- and orange-petaled flowers, two *F3′5′H* and *DFR* genes for key enzymes for petal colorization (flavonoid 3′,5′-hydroxylase and dihydroflavonol 4-reductase, respectively) were differentially expressed (Supplementary Table S2) (Sánchez-Cabrera et al., 2021).

Aberrations in flower development (including anthers) are present in cytoplasmic male sterile (CMS) plants. For instance, mitogenomes of maintainer and Ogura CMS line of cabbage contains 4 specific *orfs*, including *orf138a* and *orf154a*, whose expression led to the increased ATP production by affecting abundance of OXPHOS transcripts. In result, more energy for the abnormal proliferation of tapetal cells is produced (Zhong et al., 2021). Male sterility can be also generated by genome editing, as was shown for rice hexokinase *hxk5* mutant and for Arabidopsis knockout lines in *sarib* and *saric* genes (Lee et al., 2019; Liang et al., 2020). Knockout of *tfl1* gene in *Brassica napus* affected phase change and flowering timing (Sriboon et al., 2020).

In *C. pepo* female flowers are grown after initial male phase of development. Female flowers belong to organs with the highest number of specific genes; however, male flowers contain most differentially expressed genes. Female flower-specific genes coded proteins for cell wall biogenesis, including pectin catabolism and cell wall modification, pollen allergen Ole e 6-like (for pollen-stigma recognition) and Leu-rich extensins (pollen tube cell wall proteins), VIN3-like protein 2 - a novel TF involved in vernalization and for flowering promotion. In female flowers *flowering promoting factor 1*, some *EARLY FLOWERING* and *Ultrapetala* genes were also enriched. Male flowers specifically expressed *EPIDERMAL PATTERNING FACTOR-like protein 6*, a positive regulator of inflorescence development, *Unusual floral organs* gene for floral meristem determination and some TFs. They included male flower-specific ethylene-responsive TF 2-like from ERF family. Flowering-related genes also covered *AP-2*, *EARLY FLOWERING*, *FCA*, *FLOWERING LOCUS T* (for the florigen protein), *PISTILLATA a*nd *Flowering time control FPA-like* (Xanthopoulou et al., 2021). Among TFs regulating female flowering were NAC, ERF, bHLH, bZIP, MYB and C2H2 proteins. Interestingly, *etr1b* mutants (in the gene for one of ethylene receptors) displayed increased number of male flower nodes, indicating for the important role of *etr* genes in flowering (Segura et al., 2023). Participation of FLOWERING LOCUS T and FD TF (basic-leucine zipper TF) in flowering was investigated also in *Lemna aequinoctialis* (Yoshida et al., 2021).

Comparing with data above, distinct genes participated in the development of broccoli (*Brassica oleracea* var. *italica*) floral buds due to their morphological and anatomical specificity. The regulated DEGs between hybrid and parental cultivars coded proteins for the stress response, regulation of floral development and for the cellular signaling. In hybrid lines, DEGs for development and organ growth were affected. Overall, the upregulated DEGs coding proteins for growth and development, fatty acid and carbohydrate metabolism, protein synthesis and modifications prevailed in hybrid lines (Supplementary Table S2) (Li et al., 2018).

### Seed transcriptomes

Seed endosperm and embryo develop after double fertilization. The seed cover, on the contrary, is formed from embryo stem cells. Having reproductive function, seeds protect the embryo from external conditions (Boesewinkel and Bouman, 1984; Martín-Gómez et al., 2020).

Transcriptomic analyzes designated multiple genes active under seed biogenesis and germination. Visium Gene Expression slides (10x Genomics) allowed for the spatial profiling of various cell transcriptomes during germination of barley (*Hordeum vulgare*) seeds (Peirats-Llobet et al., 2023). Obtained results confirmed 83%–90% transcripts known from previous studies (Supplementary Table S2) (Betts et al., 2017; Shulse et al., 2019; Zhu et al., 2022). In Arabidopsis seeds, aquaporin genes were expressed in mesocotyl, scutellum and coleorhiza, and lipid-transfer protein genes - in coleorhiza. Genes for cell wall modification proteins were active in radicle and scutellum. Genes for various endonucleases, thionins, taumatins and protein inhibitors were expressed in endosperm and aleurone layer. Specific proteases were active in endosperm (Peirats-Llobet et al., 2023). Some mitochondrial PPR proteins involved in RNA processing (e.g., FLOURY ENDOSPERM22 in rice) are also necessary for the proper endosperm development (Yang et al., 2023). Recently, spatially enhanced resolution omics sequencing (Stereo-seq) and scRNA-seq used to characterize transcriptomes of germinating rice (*Oryza sativa*) embryos, led to the discovery of novel scutellum type with own genetic markers. The activity of *MTF2* gene for the mannitol transporter as well as *TG3-1*, *SCL1-2* and *CYS* genes is notable in diverse tissues of rice grains (Yao et al., 2024). Using scRNA-seq, Liew et al. (2024) showed that Arabidopsis seed embryonic cells display similar transcriptomes under germination initiation when the transcriptional remodeling occurs and cell-specific TFs affect the germination rate. In addition to scRNA-seq, snRNA-seq was used to study spatial regulation of gene expression in isolated Arabidopsis embryo nuclei, parental-embryo relationships and tissue-specific gene imprinting (Kao et al., 2021; Picard et al., 2021).

The activity of multiple genes is dynamically altered under seed development (Figures 1, 4). Under seed germination, in various embryonic cell types, DEGs for nutrient metabolism, biosynthesis, and hormone biosynthesis are particularly notable (Yao et al., 2024). Genes for ribosomal proteins, RNA-binding proteins (including RNA splicing machinery) and elongation factors are active in embryo, particularly at later stages, when transcription and translation start rapidly. Interestingly, germination without transcriptional activity belongs to specific functions of barley embryos (Betts et al., 2017). In rice, early development of seeds is characterized by the presence of SCL1-2 cell type, in later steps environmental response-like pathways are activated. Specific marker genes, like *MFT2* as well as *SAG12-1*, *CER1* and *PRP14* were activated during seed germination (Yao et al., 2024). Genes for secondary metabolite synthesis were active in the germinated seeds of *Polygonatum cyrtonema* (Liu R. et al., 2021), but not in barley grains, where it were upregulated at later stages of grain maturation (Peirats-Llobet et al., 2023). In general, highly expressed genes are enriched in Arabidopsis globular embryos and in the mature cotyledons. On the contrary, lowly expressed genes are active in the mature and post-mature stages of the green embryo, associated with dormancy transition (Figure 4) (Le et al., 2010).

During early seed imbibition, transporter genes (including mitochondrial proteins) are expressed in coleorhiza, embryo and scutellum of barley seeds. Genes for glycolytic enzymes, histone and protective protein (LEA) synthesis are also early induced in embryo; OXPHOS genes, however, are not highly expressed until the 24h-long imbibition when mitochondrial biogenesis rapidly increases. In later stages, activity of genes for aminoacid coenzyme, lipid and carbohydrate metabolism, mitochondrial transcription, nucleic acid processing and PPR proteins is notable. In some cases, genes for histone synthesis and RNA-binding proteins are active in dry seeds (Figure 4) (Narsai et al., 2017; Peirats-Llobet et al., 2023). The plastome expression is also necessary for seed development. For instance, some mTERFs (namely, mTERF2) can be targeted to chloroplasts; complete mTERF2 loss resulted in embryo lethality, but miRNA-directed knockdown of *MTERF2* gene affected chlorophyll content and plant development (Lee et al., 2021).

Multiple TFs (including bZIP, bHLH and DREB proteins) display a complex spatiotemporal expression pattern under seed development. Among 48 diverse TFs, LEC, FUS, MEDEA and PEI were notable as Arabidopsis seed development regulators. Mutations that occurred in genes for these TFs resulted in embryo defects (Le et al., 2010). Seed germination is controlled by various NAC, bZIP, DOF, HB, AP/EREBP, WRKY proteins, however genes for bZIP and AP/EREBP TFs were downregulated after seed stratification (Narsai et al., 2017; Lee et al., 2023). For early seed development, MADS TFs are also essential; the single knockout mutant (generated by genome editing) of *MADS78* or *MADS79* in rice (*Oryza sativa*) showed aberrations in endosperm biogenesis and double mutants displayed delays in seed development (Paul et al., 2020). In the cotyledon stage, SAP TFs are distinctive. The globular stage is enriched with tissue-specific ARF and WRKY TFs. Mature and postmature greened stages have common multiple, including ARR, MYB and MADS proteins. bHLH TFs are abundant in radicle, BKNs are necessary for shoot apical meristem development and bZIPs are accumulated in scutellum and endosperm. Last, but not least, for the embryo root and hypophysis development, MP and TMO TFs respectively, are necessary (Figures 2, 4) (Peirats-Llobet et al., 2023).

## Transcriptomic analyses of medicinal plant species for secondary metabolite synthesis

**FIGURE 5:**
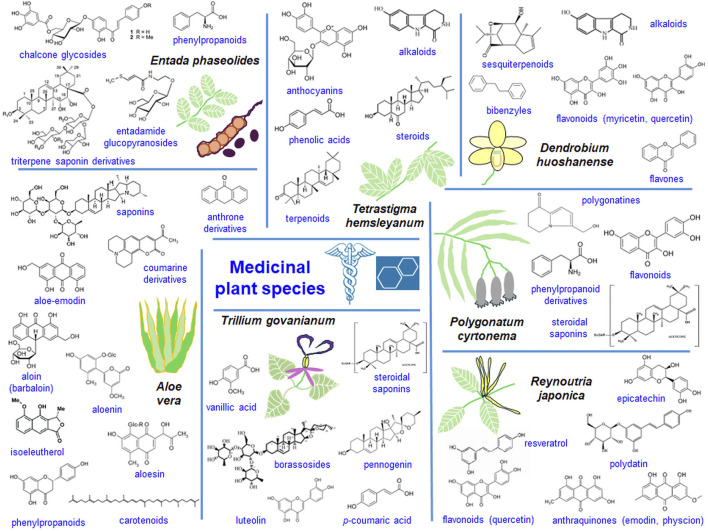
The pharmaceutical potential of selected medicinal plant species. Valuable compounds (formulas added) as secondary metabolism products of medicinal plant species were shown together with small species images.

Transcriptomic analyses of medicinal plant species allow valuable comparisons of newly discovered genes with the existing data helping to understand the complexity of secondary metabolite biogenesis from various organs and developmental stages and to determine functions of encoded biomedical proteins (Wang et al., 2020; Guo et al., 2021). The potential of transcriptomes to code proteins necessary for the synthesis of valuable biomedical compounds (Figure 5) is discussed. Details on transcriptomes of medicinal plant species are presented in Supplementary Table S2.

### Quinones, carotenoids and phytoestrogens

Aloe (*Aloe vera*) and Cape leadwort (*Plumbago auriculata*) produce of a wide range of quinone and carotenoid derivatives, including antraquinones, carotenoids and saponins (Figure 5). Numerous genes are involved in the biosynthesis of such compounds in these species (Choudhri et al., 2018; Li et al., 2024). The biosynthesis of the phenylpropanoid and terpenoid backbone is crucial for the biogenesis of plumbagin and saponin. In Cape leadwort, the biosynthesis of those compounds follows the mevalonic acid (MVA) pathway and specific “gateway enzymes” pathway. Li et al. (2024) used methyl jasmonate (MeJA) to increase the efficiency of plumbagin and saponin synthesis in *P. auriculata*. Genes for phenylpropanoid biosynthesis, cysteine and methionine metabolism, terpenoid, pantothenate, CoA and aminoacid biosynthesis were enriched among MeJa-responsive DEGs.

For the biosynthesis of environmental phytoestrogens, such as lignans with anticancer activity, flax (*Linum usitatissimum*) plants seem to be promising. To understand the relevance of lignan biogenesis for plant immunity responses, Danaeipour et al. (2023) characterized podophyllotoxin (PTOX) biogenesis pathway by flax transcriptome analysis (Supplementary Table S2).

### Terpenoids and steroids

Few terpenoids (including monoterpenes) and steroids (including steroidal saponins) are synthesized by various medicinal plant species. They are accumulated efficiently in diverse organs (terpenoids in leaves and spikes, and steroids mainly in roots). Terpenoid biogenesis is particularly complex and involves two metabolic routes: MVA and methyl-erythritol-4-phosphate (MEP) pathways that may be present in a single plant species (Ju et al., 2024). In *Trillium govanianum* genes for terpenoid biogenesis were upregulated in leaves, while genes responsible for the synthesis of steroids appeared upregulated in roots (Figure 5) (Singh et al., 2017).

Sambong (*Blumea balsamifera*), an important species used in traditional Chinese medicine, is also a source of terpenoid compounds. Ju et al. (2024) investigated transcriptomes of *B. balsamifera* leaves at various developmental stages and found 116 DEGs coding proteins for MVA and MEP terpenoid biogenesis pathways.

Tissue- and developmentally specific terpenoid biogenesis in glandular trichomes of *Nepeta tenuifolia* was investigated by Liu L. et al. (2021). Five genes for the biogenesis of *p*-menthane monoterpene were identified within the (+)-menthone pathway. In addition, functional analyzes *N. tenuifolia* limonene 3-hydroxylase (L3OH) proved its relevance for further studies (Supplementary Table S2) (Liu L. et al., 2021).

### Flavonoids, phenolic compounds and polysaccharides

Flavonoid compounds comprise flavonols (Han et al., 2023), flavone, flavonol, isoflavone and anthocyanins (Qiao et al., 2023), silymarin (Roy et al., 2018), phenylpropanoid derivatives (Liao et al., 2020), polyphenolic compounds, such as myristine and quercetin or C6C3C6-type flavonoids (Zhou et al., 2020; Yu et al., 2021) (Figure 5). Apricot fruits (*Prunus persica*) contain flavonols of antioxidant, antitumor and provascular activity. Numerous DEGs for enzymes of flavonol biogenesis were present in apricot fruit transcriptomes (Han et al., 2023). Characterization of transcriptomes from two cultivars of *Artemisia* allowed the identification of genes for the biosynthesis of flavonoids, phenylpropanoids and anthocyanins. Most genes coding enzymes for flavonoid synthesis were visibly upregulated in the ‘*NYYY*’ cultivar, which was particularly abundant in flavonoids (Qiao et al., 2023). Japanese thistle (*Cirsium japonicum*) belongs to Asiatic species with antitumor, proimmune, antidiabetic and hepatoprotective activity. It contains flavones, especially silymarin produced by the flavonoid and phenylpropanoid pathway. DEGs for flavonoid biogenesis were markedly upregulated in flowers (Roy et al., 2018). Transcriptomes of Madagascar periwinkle (*Catharanthus roseus*), important medicinal plant species, in etiolated, de-etiolated and greening cotyledons were investigated. Affected DEGs covered genes for phenylpropanoid metabolism enzymes (Supplementary Table S2) (Yu et al., 2021). Transcriptomes of leaves, roots and rhizomes of Solomon’s seal (*Polygonatum cyrtonema*), a species containing phenylpropanoids and flavonoids (Figure 5) were studied by Wang et al. (2019). Genes coding enzymes for phenylpropanoid and carbohydrate metabolism were distinctive (Wang et al., 2019).

Medicinal plant species are also a source of phenolic metabolites, including polyphenols, quinate and gallic acid derivatives, phenylpropanoids and lignins. Red alder (*Alnus rubra*) contains valuable bioactive phenolic metabolites. Hixson et al. (2024) analyzed the diverse genes coding for enzymes necessary for the synthesis of such compounds, including shikimate-chorismate-phenylalanine pathway, quinate and gallic acid derivatives, phenylpropanoid and lignin compounds, flavonoids as well as proanthocyanidins and related metabolites. Phenylpropanoid and lignin biosynthetic pathways were enriched among the studied DEGs.

Products of mevalonate, methylerythritol phosphate, shikimate and resveratrol biosynthesis pathways belongs to the most important polyphenolic compounds synthesized by medicinal plant species (Zheng et al., 2021). Japanese knotweed (*Reynoutria japonica*) contains resveratrol, flavonoids and bioactive anthraquinones, emodin and physcion, with antimicrobe and anticancer activities (Hong et al., 2016) (Figure 5). In *R. japonica* root, stem, leaf, flower and fruit transcriptomes (Supplementary Table S2) few genes coding key enzymes involved in the synthesis of resveratrol (including PAL, C4H, 4CL as well as STS/CHS synthases) were revealed (Zheng et al., 2021). Curcumin belongs to polyphenolic antioxidants used for the treatments of blood stasis and pain. Lu et al. (2020) analyzed transcriptomes of rhizomes of curcuma plants (*Curcuma wenyujin*) from two areas (Supplementary Table S2). Genes for terpene, curcumin and polysaccharide metabolism were upregulated in *C. wenyujin* from Wenzhou.

*Dendrobium huoshanense* stems are source of various alkaloids, sesquiterpenoids, flavonoids and flavones (Figure 5). In transcriptomes of leaves, stems and roots of *D. huoshanense* at least 103 genes involved in stem development and polysaccharide biosynthesis, 74 genes for glycosyltransferases and 15 genes involved in myricetin and quercetin synthesis were found (Zhou et al., 2020). Based on Meng et al. (2016) data, Shen et al. (2017) analyzed transcriptomes of flowers, leaves, roots and stems of another *Dendrobium* species, *D. officinale* (Supplementary Table S2). Between stems and roots, DEGs for proteins necessary for the metabolism of carbohydrates, pyruvate, dicarboxylates, purines and aminoacids were enriched. The potential for polysaccharide synthesis was also assessed in *Dendrobium moniliforme* (Yuan et al., 2019), where 1204 genes for carbohydrate metabolism were identified. Highly enriched genes were related to flavonoid metabolism among other categories. Another orchid species, *Bletilla striata*, is a source of various flavonoid and terpenoid compounds. The study of *B. striata* leaf, root and tuber transcriptomes allowed for finding multiple genes for metabolism of saccharides and flavonoids (Ma et al., 2022). The synthesis of methylated derivatives of flavonoids seems to be especially promising to increase their *in-planta* yield. In *Eucalyptus nitida* at least. 60 different DEGs for putative O-methyltransferases (OMTs) were selected and one of these enzymes converted flavanone pinocembrin into a methylated product in the functional test (Somaletha Chandran et al., 2022).

Among TFs regulating flavonoid biogenesis pathways were AP2-ERF, bHLH, bZIP, C2C2, TIFY, MADS, MYB, TCP, bHLH, C3H, B3, HB, E2F, GRAS, WRKY, SBP and TRAF proteins (Yu et al., 2021; Han et al., 2023; Qiao et al., 2023). TFs regulating the expression pattern of genes for polysaccharide biosynthesis included MYB, AP2-EREBP, WRKY, bHLH, zinc finger C3H and C2H2 and NAC proteins (Wang et al., 2019).

### Glycosides, anthocyanins and macrocyclic peptides

*Neopicrorhiza scrophulariiflora* accumulates picroside I and picroside II, anti-inflammatory, anti-cancer and anti-bacterial iridoid glycosides. Rao et al. (2025) identified 200 different flavonoid and >60 terpenoid compounds in this species. Among others, DEGs between investigated tissues coded proteins for the metabolism of terpenoids, polyketides and phenylpropanoids. At least 74 and 43 annotated genes coded proteins for iridoid and picroside biogenesis, respectively. Matchbox bean (*Entada phaseolides*) is a source of glycosides and glucopyranosides (Figure 5). DEGs for phenylpropanoid biosynthesis and cyanoamino acid metabolism were enriched between leaves and roots of *E. phaseolides* (Liao et al., 2020).

Medicinal plant species accumulating anthocyanins could also synthesize alkaloids, flavonoids, steroids and terpenoids (Yan et al., 2020; Ni et al., 2021). Analysis of the purple and green leaf transcriptomes of *Tetrastigma hemsleyanum*, a species with antibiotic properties, allowed identification of DEGs mainly involved in anthocyanin and carotenoid biosynthesis (Figure 5) (Yan et al., 2020). Ni et al. (2021) studied candidate genes involved in anthocyanin biosynthesis in sugarcane (*Saccharum officinarum*). The transcriptomes from rinds and piths of three cultivars of sugarcane were compared. The enriched pathways for DEGs between investigated tissues included the ones for steroid, phenylpropanoid and flavonoid biosynthesis, tryptophan metabolism, indole alkaloid biosynthesis as well as sesquiterpenoid and triterpenoid biosynthesis. At least 50 DEGs for enzymes of anthocyanin biogenesis were identified (Supplementary Table S2).

Cyclotides, which belong to stable disulfide-rich macrocyclic peptides, belong to promising drugs. Asian pigeonwings (*Clitoria ternatea*), a perennial medicinal plant species, is the only known fabaceous plant producing large amounts of cyclotides. In pods and stems of *C. ternatea* multiple cyclotide genes are highly expressed. At least 71 cyclotide precursor sequences were found, including 26 entirely novel ones. A pathway for cyclotide biogenesis, with precursor processing was also proposed (Kalmankar et al., 2020).

## Discussion

### Studies employing RNA-seq to study organ-specific and medicinal plant species transcriptomes

**FIGURE 6:**
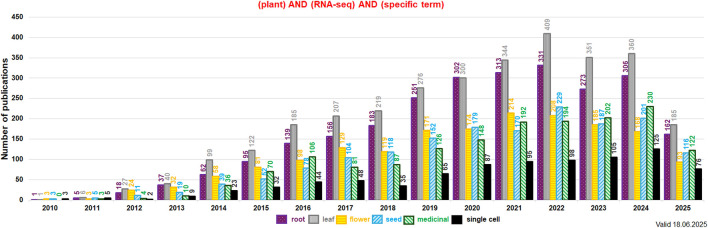
The detailed number of publications per year related to RNA-seq and investigated plant organs, medicinal plant species and single cell studies (values indicated above each bar; data for subterms indicated by the various colors and checking patterns). Keywords used in the NCBI PubMed search (https://pubmed.ncbi.nlm.nih.gov/) included: “plant,” “RNA-seq,” and the given subterm (indicated in the legend below the histogram). Data for roots, leaves, flowers, seeds, medicinal plant species and scRNA-seq were presented from 2010 onward. The analysis was performed in June 2025.

Current data on plant transcriptomes can be obtained from various studies on transcriptomes of multiple tissues, single cells or single organelles (e.g., scRNA-seq or snRNA-seq). From 2010 the number of reports on plant organ-specific transcriptomes increased notably. Studies on transcriptomes of roots and leaves dominate since 2020. Papers on generative organ and seed transcriptomes were less in quantity in this period. Recently, the number of reports on transcriptomes of medicinal plant species and single-cell transcriptomes has doubled in the last 5 years. Between 2010 and 2025 ca. 9,200 studies on root, leaf, flower and seed specific transcriptomes were published. The data for transcriptomes of medicinal plant species have been deposited in >1,600 publications between 2010 and 2025. Those numbers depicts the continuous growth of interest of plant organ transcriptomes in current plant research (Figure 6).

### Transcriptomic patterns across diverse organs

Functional terms related to the organ-specific DEGs are summarized in Figure 1. This comparison indicates the high specificity of organ transcriptomes. Genes jointly affected in diverse plant organs encode proteins participating in important activities, including hormonal signaling, aminoacid metabolism, cell organization, DNA synthesis, protein fate and intracellular transport. TFs common for all plant organs comprise mostly bHLH, MYB and NAC proteins (Figures 1, 2).

Vascularization is enriched by genes mainly involved in carbohydrate metabolism, cell divisions and cell wall reorganization. TFs for diverse aspects of vascularization processes are particularly abundant and notably vary from TFs regulating development of other plant tissues and organs (Figures 1–3).

In roots, genes coding proteins for chromatin assembly, cell wall biogenesis, cytokinesis and cytoskeleton remodeling, cell cycle, detoxication, hormonal response, DNA replication, high-energy compound catabolism, histone biogenesis, polysaccharide and phenylpropanoid synthesis, and vesicle transport are notable. Those functions likely reflect root growth, organogenesis, plasticity and responsiveness. In particular, distinct genes are active in primary and lateral roots as well as in root endodermis. Root organogenesis is regulated by multiple TFs, including the specific proteins governing development of root hairs, meristem, endodermis, primary and lateral roots (Figures 1–3).

Leaf transcriptomes particularly depends on organ polarity and organization. They are especially abundant in photosynthetic protein transcripts. Energy conversions, synthesis of secondary metabolites (including wax), various morphogenic functions, protein assembly, redox homeostasis belong to main functions of leaf-affected genes. Various TFs are involved in the organogenesis of leaf tissues or morphological zones, however AHL, bHLH, MYB and WRKY TFs overlaps between them. In senescing leaves genes for autophagy and diverse compound degradation are accompanied by transcriptomic alterations leading to the rearrangement of organellar biogenesis; distinct TFs are also active (Figure 3).

Flower transcriptomes are enriched with the activity of genes for the vegetative to generative phase shift as well as with genes for stimulus detection, fatty acid and carbohydrate metabolism, floral meristem identity, hormonal signaling, pigment synthesis, photoperiod regulation, pollen development and production of secondary metabolites. The activity of various TFs is necessary for flower development, including specific TFs for the pigment biogenesis (Figures 1, 2).

Compared to vegetative organs, seeds have quite distinct transcriptomes. They code proteins for water homeostasis, transcription and translation machinery, protective and transporter activities and for starch metabolism. Seed transcriptome is in a very complex manner remodeled in various tissues during seed development, imbibition and germination (with notable mobilization of organellar biogenesis in those processes). Seeds contain also a vast number of specific TFs, which are differentially switched on during seed maturation (Figures 1, 2, 4).

### Future directions and concluding remarks

In this review, organ-specific alterations from a number of studies on plant model and crop species were analyzed with a focus on mostly affected protein genes including genes for organ-specific TFs. The involvement of multiple marker genes and genes coding for organellar proteins in plant development was also highlighted. In general, under plant organogenesis, organ-specific genes are switched on and off in a highly complex, spatiotemporal manner.

From the variety of studied species, it is obvious that basic transcriptomic studies should be expanded to less-studied model, crop and medicinal plant species, which produce a particularly wide range of valuable secondary metabolites (Yan et al., 2020; Ju et al., 2024; Li et al., 2024). All future research should be also validated in field conditions, because transcriptomic patterns vary from controlled to field grown conditions and the production of medicinal compounds in natural conditions can be more effective.

In addition, modern methodologies including gene editing and advanced gene engineering should be broadened to allow (1) to study the relevance of additional marker genes or mainly affected organ-specific DEGs under plant development and (2) to increase the potential of secondary metabolite synthesis. Gene editing could particularly be used for precise plant breeding by (1) knock-out of genes negatively affecting yield or useful agronomical traits, (2) knock-in and gene replacement to introduce new alleles or to decrease trait multiplicity and to modify promoters or coding sequence in developmentally important genes. Those attempts may be used to improve the crop yield or to modify plant phenotype (Chen et al., 2019). For instance, tomato (*Solanum lycopersicum*) lines with the edited *SlCHRC* gene (coding for carotenoid binding protein regulating chromoplast development in fruits) displayed greener phenotype with lowered carotenoid and plastoglobuli content, contrary to lines overexpressing this protein (Wang et al., 2024a). By virus-induced gene silencing (VIGS), Huang et al. (2022) knocked-down cotton (*Gossypium hirsutum*) genes for Vir-like m^6^A methyltransferase associated proteins which affected plastid-dependent and independent leaf development by altering expression of multiple target genes. In this review some other examples of editing of developmentally important genes were also presented (Wang et al., 2017; Lee et al., 2019; Liang et al., 2020; Paul et al., 2020; Sriboon et al., 2020; Liu et al., 2024). In addition, Cao et al. (2024) proposed a simple transformation and gene editing protocol which should be prospective for numerous medicinal plant species. It employs *Agrobacterium*- mediated root transformation for the delivery of editing reagent.

Key metabolic pathways of pharmaceutical importance should be more effectively investigated in medicinal plant species. Obviously, further studies are awaited to understand better how transcriptomic analyses can improve synthesis of natural products in plant-based bioreactors. Those attempts are needed (1) to scale up metabolite synthesis, (2) to synthesize more modified or novel compounds and (3) to optimize parallel synthesis of multiple compounds for obtaining more efficient yield. The ability of the synthesis of selected compounds can be, however, hampered by the insufficient transcriptomic data (Kalmankar et al., 2020; Danaeipour et al., 2023; Rao et al., 2025) which should be improved in terms of complexity.

## Glossary

ABC: ATP-Binding cassette
ABI/VP: ABA-INSENSITIVE/VIVIPAROUS
ACL: ATP-Citrate lyase
AGL: AGAMOUS LIKE
AHL: AT-HOOK MOTIF NUCLEAR LOCALIZED PROTEIN 23
AIL: AINTEGUMENTA-LIKE
ANAC: NAC Domain containing protein
ANT: AINTEGUMENTA
AP: APETALA
APL: ALTERED PHLOEM DEVELOPMENT
ARF: auxin responsive factor
ARG: arginase
ARR: authentic response regulator
ATAF: *Arabidopsis thaliana* activating factor
ATHB: small homeodomain-leucine zipper family
ATL: ARABIDOPSIS TOXICOS EN LEVADURA
AUX/IAA: auxin/indole-3-acetic acid
B3: B3 DNA-binding domain
BAM: receptor kinase
BELL: BEL1-like
BFN: bifunctional nuclease
bHLH: basic helix–loop–helix
BIM: BES1-INTERACTING MYC-LIKE, a bHLH transcription factor
BKN: barley knox
BSK: BRASSINOSTEROID-SIGNALING KINASE
bZIP: basic (region) leucine zipper
CAMTA: calmodulin binding transcription activator
CBF: C-repeat binding factor
C2C2: TF with zinc finger
CCOAOMT: caffeoyl-CoA O-methyltransferase
CCR: cinnamoyl-CoA:NADP reductase
CDK: cyclin-dependent kinase
CER: ECERIFERUM
C2H2: cysteine 2/histidine 2
C3H: cysteine 3/histidine
C4H: cinnamate 4-hydroxylase
CHRC: chromoplast-specific carotenoid-binding protein
CHS: chalcone synthase
4CL: 4-coumarate: CoA ligase
CLE: CLAVATA3/ESR-RELATED
CMS: cytoplasmic male sterility
CNA: CORONA
CO: CONSTANS
COBL: COBRA-LIKE
COX: cytochrome *c* oxidase
CRY: cryptochrome
CUC: cup-shaped cotyledon
CYS: cystatin
cyt.: cytochrome
DEG: differentially expressed gene
DFR: dihydroflavonol 4-reductase
DMAPP: dimethylallyl pyrophosphate
DOF/Dof: DNA-binding with one finger
DREB: dehydration-responsive element-binding
EF: early flowering
E2F: transcription factor binding DNA through the E2 recognition site
ELP: EXTENSIN-LIKE PROTEIN
EREBP: ethylene-responsive element binding protein
ERF: ethylene response factor
ETR: ethylene receptor
FAMA: a regulator of stomatal development, TF
FAR: FAR-RED IMPAIRED RESPONSE
FCA: FLOWERING LOCUS CA
FD: FLOWERING LOCUS D
FE: ALTERED PHLOEM DEVELOPMENT, a phloem-specific Myb-related protein
F3′5′H: flavonoid 3′,5′-hydroxylase
FIE: fertilization-independent endospermia
FKF: FLAVIN-BINDING KELCH REPEAT F-BOX
FMA: FAMA, A BHLH TRANSCRIPTION FACTOR
FPA: FLOWERING PATHWAY
FUL: FRUITFUL
FUS: fused in sarcoma
G2: Golden 2
GATA: (T/A)GATA(A/G)-binding transcription factors
GER: GERMIN
GH: glycosyl hydrolase
GI: GIGANTEA
GID: GIBBERELLIN-INSENSITIVE DWARF
GL: GLABRA2
GO: gene ontology
GPAT: glycerol-3-phosphate acyltransferase
GPI: glycosylphosphatidylinositol
GRAS: GIBBERELLIN-ACID INSENSITIVE/REPRESSOR of GA1/SCARECROW
GRF: growth regulating factor
HAN: HANABA TANARU
HAP: transcription factor complex
HAT: histone acetylases
HB: homeobox
HCT: hydroxycinnamoyl transferase
HD: homeodomain
HDT: histone deacetylase
HLH: helix-loop-helix
HLZ: Homeobox-Leucine Zipper
HPLC: high pressure liquid chromatography
HSF: heat shock factor
hxk: hexokinase
IFL: INTERFASCICULAR FIBERLESS
IPP: isopentenyl pyrophosphate
IRX: IRREGULAR XYLEM
JA: jasmonic acid
KAN: KANADI
KCS: 3-ketoacyl-CoA synthase
KNAT: KNOTTED-like homeobox of *ARABIDOPSIS THALIANA*
KNOX: KNOTTED1-like homeobox
LAC: laccase
LACS: long-chain acyl-CoA synthetase
LBD: LATERAL ORGAN BOUNDARIES DOMAIN
LEA: late-embryogenesis abundant
LEC: little elongation complex
Lhc: light-harvesting complex
LHY: Late Elongated Hypocotyl
L3OH: limonene 3-hydroxylase
LRL: ROOTHAIRLESS LIKE
LRS: lateral roots
MADS: MINICHROMOSOME MAINTENANCE 1/AGAMOUS/DEFICIENS/SERUM RESPONSE FACTOR
MEDEA: motif enrichment in differential elements of accessibility
MEE: MATERNAL EFFECT EMBRYO ARREST
MeJA: methyl jasmonate
MEP: methyl-erythritol-4-phosphate
MIF: mini zinc-finger
miRNA: micro-RNA
MP: MONOPTEROS
mTERF: mitochondrial transcription termination factor
MTF: metal regulatory transcription factor
MTF2: mannitol transporter protein
MYB: myeloblastosis
MVA: mevalonic acid
NAC: no apical meristem/ATAF1/cup-shaped cotyledon
Nam: no apical meristem
NCED: 9-cis-epoxycarotenoid dioxygenase
ncRNA: non-coding RNA
NDH: NADH dehydrogenase, chlororespirating
NF-Y: nuclear transcription factor Y
NYE: NON-YELLOWING
NPY: NAKED PINS IN YUC MUTANTS
Ole e: allergen from the pollen of olive tree (*Olea europaea*)
OMT: O-methyltransferase
ORF/orf: open reading frame
OXPHOS: oxidative phosphorylation
PA: phloem-associated
PAL: phenylalanine ammonia lyase
PASPA: PUTATIVE ASPARTIC PROTEINASE A3
PcG: Polycomb group
PEAR: PHLOEM EARLY DNA-BINDING-WITH-ONE-FINGER
PEI: Cys3His zinc finger domain-containing protein
PER: peroxidase
PHB: PHABULOSA
PHD: plant homeodomain
PHV: PHAVOLUTA
PHY: phytochrome
PIN: PINFORMED
PLT: PLETHORA
PRR: PSEUDO RESPONSE REGULATOR
PSB/psb: photosystem II subunits
PXE/PXY: PHLOEM INTERCALATED WITH XYLEM
PTOX: podophyllotoxin
RBCS: Rubisco small subunit
RCP: reduced carotenoid pigmentation/ROOT CAP
REV: REVOLUTA
RNA-seq: RNA sequencing
RPL: ribosomal large subunit
RPS: ribosomal small subunit
RSI: ROSINA
SAP: STERILE APETALA
SAG: senescence-associated gene
SBP: SQUAMOSA Promoter Binding Protein
SCL: scutellum epidermis/layer
SCR: SCARECROW
scRNA-seq: single cell RNA sequencing
SEO: sieve element occlusion
SHR: SHORT ROOT
siRNA: small interfering RNA
SMB: SOMBRERO
SMXL: SUPPRESOR OF MAX-LIKE
SNF: sucrose non-fermentable
snRNA-seq: single nucleus RNA sequencing
SOC: suppressor of overexpression of *constans*
STH: saitohin
STS: stachyose synthase
SUC: SUCROSE TRANSPORTER
Tat: twin-arginine translocation
TCA: tricarboxylic acid cycle
TCP: TEOSINTE BRANCHED1/CYCLOIDEA/PROLIFERATING CELL FACTOR
TG3-1: HYBRID PROLINE- OR GLYCINE-RICH PROTEIN 5
TF: transcription factor
tfl: terminal flowering
TIFY: conserved core motif TIF[F/Y]XG
TLP: Tubby-like protein
TMO: TARGET OF MONOPTEROS
TRAF: Tumor necrosis factor receptor-associated factors
VIGS: virus-induced gene silencing
VIN: VERNALIZATION INSENSITIVE
VND: VASCULAR-RELATED NAC-DOMAIN
WER: WEREWOLF
WGD: whole-genome duplication
WOL: WOODEN LEG
WOX: WUSCHEL-Related Homeobox
WRKY: transcription factor family
XCP: xylem peptidase
ZIP: leucine zipper
